# Supplementary material for: Single-cell RNA sequencing analysis of human Alzheimer’s disease brain samples reveals neuronal and glial specific cells differential expression
Source: PLoS One. 2023 Feb 24;18(2):e0277630. doi: 10.1371/journal.pone.0277630 (PMC9955959; doi:10.1371/journal.pone.0277630)

Graphical Abstract

Single-cell sequencing of Alzheimer's disease (AD) and control brain samples

A. Cell-partitioning

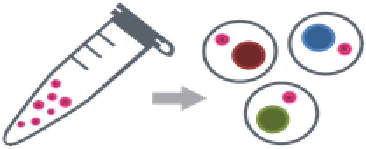

B Library Preparation

C Single cell RNA Sequencing (Chromium)

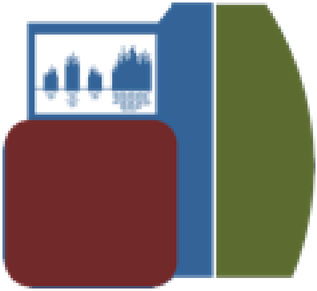

D Computational Data Analysis

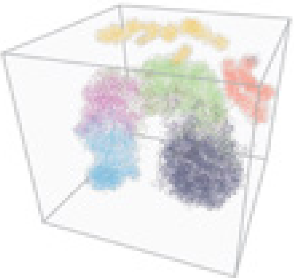

Supplement: S1 Graphical abstract — (PDF) [file pone.0277630.s001.pdf]
