# Supplementary material for: Single-cell RNA sequencing analysis of human Alzheimer’s disease brain samples reveals neuronal and glial specific cells differential expression
Source: PLoS One. 2023 Feb 24;18(2):e0277630. doi: 10.1371/journal.pone.0277630 (PMC9955959; doi:10.1371/journal.pone.0277630)
Supplement: S1 File — (PDF) [file pone.0277630.s004.pdf]

**Material Transfer Agreement for the Supply of Human Tissue Materials FOR USE where the material is human organs, tissue or cells (other than human gametes or embryos) but NOT where the intended use is transplantation or human application**

This Agreement is made by and between:

a) University of Edinburgh, Old College, South Bridge, Edinburgh, EH8 9YL

and

b) <*Name of Recipient Scientist's Institution and address*> ("the Recipient Institution")

This Agreement records the terms under which the Provider Institution will make available to the Recipient Institution the Material identified in Appendix 2 (the "Material"). The term "Material" means material, other than human gametes or embryos, which consists of, or includes human cells and which is considered "Relevant Material" for the purposes of the Human Tissue Act 2004[1] together with related data. The Recipient Institution will hold the Material on the terms of this Agreement and solely for the purpose of Neurocytopathology associated with ageing ("the Study") and as described in Appendix 1, within the research group of Dr Rickie Patani ("the Recipient Scientist"). The Recipient Institution hereby agrees to comply and procure that the Recipient Scientist and all personnel who work with the Material comply with the following terms and conditions:

1. The Recipient Institution will not use the Material for administration to human subjects or human application as that term is defined in the Human Tissue (Quality and Safety for Human Application) Regulations 2007 (or equivalent as each may be replaced or amended from time to time), or for clinical or diagnostic purposes.[2]

2. The Recipient Institution may use the Material for the purposes of the Study and as described in Appendix 1, from the date of receipt of the Material. The Recipient Institution will comply fully with all applicable environmental, health and safety laws, the Human Tissue Act 2004 and other Applicable Laws[3] with respect to its use (including, but not limited to, disposal or return).

[1] The Human Tissue Act 2004 applies to the "authorised activities" principally the removal, storage and use of "Relevant Materials" (as defined under the Act, including human cells, tissue and organs, but not cell lines) which come from a living or deceased person for "Scheduled Purposes" (these are set out in Schedule 1 of the Act, including, but not limited to, "research in connection with disorders, or the function of the human body", "education or training relating to human health", and "transplantation").

[2] The Human Tissue (Quality and Safety for Human Application) Regulations 2007 apply to the procurement, testing, processing, storage, distribution, and import or export of tissues and cells (including cell lines). "Cells" mean human cells (whether individually or in an unbound collection) including cell lines, but not including gametes, embryos outside the body, blood or blood components. "Tissue" for the Regulations, means all constituent parts of the human body formed by cells, but not including gametes and embryos outside the body (which are regulated by the Human Fertilisation and Embryology Authority pursuant to the Human Fertilisation and Embryology Act 1990), or organs.

[3] Applicable Laws means all laws, rules, regulations, codes of practice, research governance or ethical guidelines, or other requirements of any Regulatory Authority, that may apply to the use of the Material by the Recipient Institution from time to time, including (but not limited) the Human Tissue Act 2004 or the Human Tissue (Scotland)_Act 2006, the Human Tissue (Quality and Safety for Human Application) Regulations 2007, the Human Fertilisation and Embryology Act 1990 (as amended), the EU Tissues and Cells Directive (2004/23/EC) and

3. The Recipient Institution shall use a courier with suitable skill and experience to safely transport the Material in accordance with all Applicable Laws. The Recipient Institution will bear the cost of carriage and any necessary insurance. The Provider Institution makes no charge for the Material / the Material is provided subject to the reimbursement by the Recipient Institution to the Provider Institution for its costs of extracting from storage and preparing the Material as set out in Appendix 2. Risk in and responsibility for the Material shall pass to the Recipient Institution once it is loaded onto transport as organised by the Recipient Institution. If so requested by the Provider Institution the Recipient Institution shall provide it with written confirmation of the safe receipt of the Materials promptly after their delivery to the Recipient Institution's laboratory.

4. The Recipient Institution understands that the Material may have hazardous properties, contain infectious agents or pose other health and safety risks. Subject to clause 9, the Provider Institution makes no representations and gives no warranties either express or implied in relation to it: for example (without limitation), no warranties are given about quality or fitness for a particular purpose, or freedom from infection. The Provider Institution will not be liable for any use made of the Material by the Recipient Institution. The Recipient Institution will use the Material in accordance with good laboratory practice standards, all due skill and care and with dignity, sensitivity and respect. The Recipient Institution will comply with all Applicable Laws, approvals, rules, codes of practice and regulations governing the transportation, storage, use and disposal of the Material. The Recipient Institution warrants that it will only use, or permit the use of the Material in work that has ethical approval, as stated in Appendix 1.

5. Except to the extent prohibited by Law and subject to clause 9, the Recipient Institution assumes all liability for damages which may arise from its receipt, use, storage or disposal of the Material. The Provider Institution will not be liable to the Recipient Institution for any loss, claim or demand made by the Recipient Institution, or made against the Recipient Institution by any other party, due to or arising from its use, storage or disposal of the Material by the Recipient Institution, except to the extent the law otherwise requires.

6. The liability of either party for any breach of this Agreement, or arising in any other way out of the subject matter of this Agreement, will not extend to loss of business or profit, or to any indirect or consequential damages or losses.

7. The Recipient Institution agrees to obtain the written consent of the Provider Institution if there is any material change to the proposed use of the Material in the Study as described in Appendix 1.

Commission Directives 2006/17/EC and 2006/86/EC. The Human Tissue Authority Directions and Codes of Practice, and the Medicines for Human Use (Clinical Trials) Regulations 2004, as updated and amended from time to time and, where relevant, the national implementations of the same.

8. The Recipient Scientist will acknowledge the source of the Material in any publication reporting on its use. If the Recipient Scientist wishes to include in a publication any information which has been provided by the Provider Institution with the Material and which was clearly marked as “confidential” and “proprietary” at the point of disclosure (“Confidential Information”), the Recipient Scientist must obtain written permission from the Provider Institution, providing a copy of the text to allow a reasonable period for review before publication takes place, such permission not to be unreasonably withheld or delayed. If so requested by the Provider Institution, the Recipient Institution shall provide the Provider Institution with a confidential copy of the findings of the Study.

9. The Provider Institution warrants that where required by Applicable Laws the Material has been obtained from humans with the appropriate consent as required by the Human Tissue Act 2004 and with ethical approval and the Provider Institution shall be liable for any claims arising due to the breach of this warranty. The Provider Institution hereby grants to the Recipient Institution a non-exclusive research licence to use the Material for the Study only. The Provider Institution further warrants that it has not provided any information (and does not intend to provide any information) which has led or may lead to the Recipient Institution being able to identify the person from whom the relevant material came.

10. The Recipient Institution undertakes to store the Material in accordance with all Applicable Laws and not to attempt to identify or contact the donor of the Material or to compromise or otherwise infringe the confidentiality of information on the donors and their right to privacy.

11. Nothing included in this Agreement shall prevent the Provider Institution from being able to distribute the Material to other entities as described in Appendix 1. If, as per the details included in Appendix 1, the Material is to be transferred to another institution for the purposes of the Study, the responsibility for compliance with the terms of this Agreement rests with the Recipient Institution.

12. The Provider Institution has the right to terminate this agreement forthwith at any time by means of written notice to Recipient Institution if the ethical approval is withdrawn or if the Recipient Institution is in breach of this Agreement. In the case of any termination, the Recipient Institution shall immediately discontinue all use of the Material and, at the Provider Institution's discretion, promptly return or destroy (at the Recipient Institution's own cost) all unused Material and provide written confirmation that this has been completed. If requested, the Recipient Institution must certify that it has complied in full with any such requirement of the Provider Institution. Should an individual donor or their next of kin rescind their consent, the Provider Institution will require and the Recipient Institution agrees to discontinue using the appropriately identified sample and return or destroy it in accordance with the Provider Institution’s instructions.

13. This Agreement shall be governed by English Law, and the English Courts shall have exclusive jurisdiction to deal with any dispute which may arise out of or in connection with this Letter Agreement.

14. **We require that both the unique identifying number (SD, or RU, or UA) and the unique MRC data base number (BBN) be provided as supplementary material of any publication.**

| | |
|---|---|
| Accepted and Agreed *by an authorised signatory* on behalf of | Accepted and Agreed on behalf of |
| University of Edinburgh | *<the Recipients Institution>* |
| Name: Colin Smith | Name: Dr Rickie Patani |
| Position: Reader in Pathology | Position: Wellcome Trust Clinician Scientist |
| Signature | Signature: 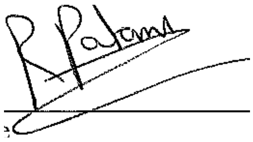 |
| Date: | Date: 20/06/2015 |

## APPENDIX 1: Study description and details of Materials

***TO BE COMPLETED BY THE RECIPIENT INSTITUTION'S SCIENTIST:***

**1. STUDY DESCRIPTION:**

*Neurocytopathology associated with ageing*

**2. DETAILS OF MATERIALS REQUESTED (type of material, quantity, numbers of material):**

*x3 young and x3 older control cases. SD033/08, SD005/09, SD006/09, SD007/09, SD026/09, SD023/10 Frozen tissue from frontal cortex (BA9), central white matter and cerebellum.*

**3. DETAILS OF COURIER TO BE USED AND COURIER ACCOUNT CODE:**

*Transported by myself (Rickie Patani) by train*

**4. LOCATION OF LABORATORY WHERE MATERIALS ARE TO BE HELD/USED:**

Department of Molecular Neuroscience,
UCL Institute of Neurology
Queen Square, London, WC1N 3BG, UK

**5. HTA LICENCE / ETHICS APPROVAL:**

**Complete one of the following:**

Where the Materials are supplied by the Provider Institution from a research tissue bank which may be a diagnostic archive and which has been granted REC approval for specific research projects, this REC approval may cover the research Study with the materials at the Recipient Institution. If this is the case, the Designated Individual (or their duly authorised delegate) of the Provider Institution confirms that its REC approval for the tissue bank will cover the proposed Study by signing here:

**Committee: East of Scotland Research Ethics Service (EoSRES)**

**Ref: 11/ES/0022**

____________________

……………………………………………

<u>**APPENDIX 2: Delivery and Storage of Materials**</u>

**TO BE COMPLETED BY THE PROVIDER INSTITUTION:**

1. **QUANTITY OF MATERIALS TO BE DELIVERED:**

**TR30/15** Approx. 0.5g

| Case No | Area | Frozen |
|---|---|---|
| SD023/10 | CWM | ✓ |
| | CB | ✓ |
| | FPS | |
| SD026/09 | CWM | ✓ |
| | CB | ✓ |
| | FPS | ✓ |
| SD007/09 | CWM | ✓ |
| | CB | ✓ |
| | FPS | ✓ |
| SD006/09 | CWM | ✓ |
| | CB | ✓ |
| | FPS | ✓ |
| SD005/09 | CWM | ✓ |
| | CB | ✓ |
| | FPS | ✓ |
| SD033/08 | CWM | ✓ |
| | CB | ✓ |
| | FPS | |

**2. COST OF SAMPLE PREPARATION:** Nil. The recipient will be responsible for the transportation

**3. CONDITIONS OF STORAGE;** As per study requires

**4. RETURN/DESTRUCTION OF SURPLUS MATERIALS ON COMPLETION OF STUDY**

If there are any Materials left over from the Study, the Recipient Institution needs to provide confirmation to the Provider Institution that any remaining Material will be destroyed (and if destroyed the Recipient Institution needs to provide confirmation to the Provider Institution that this has been completed).
