# Supplementary material for: Single-cell RNA sequencing analysis of human Alzheimer’s disease brain samples reveals neuronal and glial specific cells differential expression
Source: PLoS One. 2023 Feb 24;18(2):e0277630. doi: 10.1371/journal.pone.0277630 (PMC9955959; doi:10.1371/journal.pone.0277630)
Supplement: S2 File — IRAS project ID 199294. (PDF) [file pone.0277630.s005.pdf]

East of Scotland Research Ethics Service (*EoSRES*)

TAyside medical Science Centre
Residency Block Level 3
George Pirie Way
Ninewells Hospital and Medical School
Dundee DD1 9SY

Professor Colin Smith
NHS Lothian
Academic Neuropathology, CCBS
Chancellor's Building
Little France,
Edinburgh
EH16 4SB

Date: **11 October 2016**
Your Ref:
Our Ref: AG/16/ES/0084
Enquiries to: Arlene Grubb
Direct Line: 01382 383848

Dear Professor Smith

**Title of the Research Tissue Bank:**	**Edinburgh Brain Bank**
**REC reference:**	**16/ES/0084**
**Designated Individual:**	
**IRAS project ID:**	**199294**

Thank you for your letter of 4 October 2016, responding to the Committee's request for further information on the above research tissue bank and submitting revised documentation.

The further information has been considered on behalf of the Committee by the Chair.

We plan to publish your research summary wording for the above study on the HRA website, together with your contact details. Publication will be no earlier than three months from the date of this favourable opinion letter. The expectation is that this information will be published for all studies that receive an ethical opinion but should you wish to provide a substitute contact point, wish to make a request to defer, or require further information,, please contact the REC Manager Mrs Arlene Grubb, eosres.tayside@nhs.net. Under very limited circumstances (e.g. for student research which has received an unfavourable opinion), it may be possible to grant an exemption to the publication of the study.

Confirmation of ethical opinion

On behalf of the Committee, I am pleased to confirm a favourable ethical opinion of the above research tissue bank on the basis described in the application form and supporting documentation as revised.

The Committee has also confirmed that the favourable ethical opinion applies to all research projects conducted in the UK using tissue or data supplied by the tissue bank, provided that the release of tissue or data complies with the attached conditions. It will not be necessary for these researchers to make project-based applications for ethical approval. They will be deemed to have ethical approval from this committee. You should provide the researcher with a copy of this letter as confirmation of this. The Committee should be notified of all projects receiving tissue and data from this tissue bank by means of an annual report.

This application was for the renewal of a Research Tissue Bank application. The previous REC Reference number for this application was 11/ES/0022.

Additional conditions of approval

In addition to the standard conditions attached, ethical approval is subject to the following:

You should notify the REC once all conditions have been met (except for site approvals from host organisations) and provide copies of any revised documentation with updated version numbers. Revised documents should be submitted to the REC electronically from IRAS. The REC will acknowledge receipt and provide a final list of the approved documentation for the study, which can be made available to host organisations to facilitate their permission for the study. Failure to provide the final versions to the REC may cause delay in obtaining permissions.

Duration of ethical opinion

The favourable opinion is given for a period of five years from the date of this letter and provided that you comply with the standard conditions of ethical approval for Research Tissue Banks set out in the attached document. You are advised to study the conditions carefully. The opinion may be renewed for a further period of up to five years on receipt of a fresh application. It is suggested that the fresh application is made 3-6 months before the 5 years expires, to ensure continuous approval for the research tissue bank.

Research Tissue Bank Renewals

The Research Tissue Bank has been renewed for a further five years from the end of the previous five year period. The previous study ran for a five year period from 3 November 2011.This Research Tissue Bank may be renewed for further periods of five years at a time by following the process described in the above paragraph.

Approved documents

The documents reviewed and approved at the meeting were:

**Document**	**Version**	**Date**
Covering letter on headed paper [Cover letter]		25 May 2016
Covering letter on headed paper [Cover letter]	1	04 October 2016
Human Tissue Authority licence [NHS Lothian tissue governance support]		09 May 2016
IRAS Checklist XML [Checklist_07062016]		07 June 2016
IRAS Checklist XML [Checklist_04102016]		04 October 2016
IRAS Checklist XML [Checklist_05102016]		05 October 2016
Other [MS PIS back]	1	30 September 2016
Other [Dementia Pre-mortem consent form]	7	30 September 2016
Other [CV]	12	30 September 2016
Other [EBB Controlled document list]	2	05 October 2016
Participant consent form [LINCHPIN PCF]	7	30 September 2016
Participant consent form [MND PCF]	7	30 September 2016
Participant consent form [Generic pre-mortem consent]	7	30 September 2016

Participant information sheet (PIS) [MS PIS]		
Participant information sheet (PIS) [MND PIS]	3	21 March 2011
Participant information sheet (PIS) [MS PIS]	1	30 September 2016
Participant information sheet (PIS) [Generic patient information]	8	30 September 2016
Participant information sheet (PIS) [Dementia PIS]	3	30 September 2016
Participant information sheet (PIS) [LINCHPIN PIS]	6	05 October 2016
Protocol for management of the tissue bank [Protocol for brain bank management]	2	
REC Application Form [RTB_Form_07062016]		07 June 2016
Relative consent form [SD relative consent form]	6	30 September 2016
Relative information sheet [SD relative information sheet]	7	30 September 2016
Relative information sheet [Dementia relative information sheet]	4	05 October 2016
Summary of research programme(s) [Research Summary]	2	20 April 2016

Licence from the Human Tissue Authority

Thank you for providing a copy of the above licence.

Research governance

A copy of this letter is being sent to the R&D office responsible for NHS Lothian.

You are advised to check their requirements for approval of the research tissue bank.
Under the Research Governance Framework (RGF), there is no requirement for NHS research permission for the establishment of research tissue banks in the NHS. Applications to NHS R&D offices through IRAS are not required as all NHS organisations are expected to have included management review in the process of establishing the research tissue bank.

Research permission is also not required by collaborators at tissue collection centres (TCCs) who provide tissue or data under the terms of a supply agreement between the organisation and the research tissue bank. TCCs are not research sites for the purposes of the RGF.

Research tissue bank managers are advised to provide R&D offices at all TCCs with a copy of the REC application for information, together with a copy of the favourable opinion letter when available. All TCCs should be listed in Part C of the REC application.

NHS researchers undertaking specific research projects using tissue or data supplied by a research tissue bank must apply for permission to R&D offices at all organisations where the research is conducted, whether or not the research tissue bank has ethical approval.

Site-specific assessment (SSA) is not a requirement for ethical review of research tissue banks.

Statement of compliance

The Committee is constituted in accordance with the Governance Arrangements for Research Ethics Committees and complies fully with the Standard Operating Procedures for Research Ethics Committees in the UK.

After ethical review

Reporting requirements

The attached standard conditions give detailed guidance on reporting requirements for research tissue banks with a favourable opinion, including:

- Notifying substantial amendments
- Submitting Annual Progress reports

The HRA website also provides guidance on these topics, which is updated in the light of changes in reporting requirements or procedures.

User Feedback

The Health Research Authority is continually striving to provide a high quality service to all applicants and sponsors. You are invited to give your view of the service you have received and the application procedure. If you wish to make your views known please use the feedback form available on the HRA website: http://www.hra.nhs.uk/about-the-hra/governance/quality-assurance/

HRA Training

We are pleased to welcome researchers and R&D staff at our training days – see details at http://www.hra.nhs.uk/hra-training/

**16/ES/0084**	**Please quote this number on all correspondence**

Yours sincerely

Arlene Grubb

For Dr Robert Rea
Chair

E-mail: eosres.tayside@nhs.net

Enclosures: Standard approval conditions [SL-AC3]
