# Supplementary material for: Single-cell RNA sequencing analysis of human Alzheimer’s disease brain samples reveals neuronal and glial specific cells differential expression
Source: PLoS One. 2023 Feb 24;18(2):e0277630. doi: 10.1371/journal.pone.0277630 (PMC9955959; doi:10.1371/journal.pone.0277630)
Supplement: Attachment — Submitted filename: rebattle_letter_04_08_LS.docx [file pone.0277630.s006.docx]

04/08/22

Re-battle letter (PLoS ONE) Soreq L et al

Paper title **Single cell RNA sequencing analysis of human Alzheimer’s disease brain samples reveal neuronal and glial specific cells differential expression**

Reviewer 1

Comment: The manuscript must describe a technically sound piece of scientific research with data that supports the conclusions. Experiments must have been conducted rigorously, with appropriate controls, replication, and sample sizes. The conclusions must be drawn appropriately based on the data presented.

Partly

Reply: Yes, the paper is technically sound the RNA was extracted from the post mortem samples in high quality and the Chromium Seq experiment was done in high standard. The experimental data supports the conclusions. The manuscript describes a technically sound piece of scientific research with data that supports the conclusions. Experiments have been conducted rigorously, with appropriate controls, replication, and sample sizes (n=4 in this case). The conclusions are drawn appropriately based on the data presented.

Comment: The authors compare their data to available datasets from mouse and human, so a comparison of brain region specific data should also be included.

Reply: Indeed this was the case, in fact comparison also to cell type specific mouse brain RNASeq dataset was done (brain-map.org) and in fact we are not aware of any other available brain region specific seq publicly available data as for the time of paper submission (human brain RNA expression data and in fact mice as well is still quiet rare, in particular large datasets). But we did analyze we have analysed the GEO dataset GSE157827 (composed of a total of 21 samples, 13 Alzheimer’s and 9 neurological controls (figure 6).

Comment: According to the methods section both AD cases were male, while the control cases were male and female. Although the study is not powered to detect gender specific differences this should at least be commented on, as more and more evidence emerges that sex differences with respect to AD related changes are profound.

Reply: Apologize both AD samples were males (marked in blue in ST1), indeed we are aware of gender related expression differences in RNA expression values this is why we included only males in the current study.

5. Throughout the manuscript there are many incomplete or duplicated sentences as well as typos. For example on page 4: "...response in human vs mice MG depleted mice..." or on page 12, where the whole sentence referring to the value of the resource for the scientific community appears to be duplicated. This hampers the overall readability of the manuscript.

Reply: Thank you for this comment. We went over the paper text thoroughly removed duplicates and corrected spelling/grammar were needed.

Reviewer #2: In this study, the authors identify transcriptional networks impacted into distinct neuronal populations in human Alzheimer’s disease and control brains. Although the number of brains is small, the strength is that the authors surveyed gene expression differences in over 25,000 single-nuclei collected from the brains of disease patients in Braak stage I and II hippocampal brain samples. This study is timely and will be of interest to a large readership involved in neurodegenerative disease research and, more largely, in molecular neurosciences.

Reply: Thank you.

Major concern:

A major challenge is to understand the functional significance of gene expression differences observed in human neurodegenerative disease conditions. Although some of these differences may be disease specific, some others may be shared across disease conditions, particularly if they correspond to homeostatic responses. Here, the comparison of mouse and human data is needed. To address the gap between gene expression data and functional data, the authors may want to compare their data with functional data obtained in mouse and invertebrate models of neurodegenerative disease insults as, should overlaps be detected, this might add much insight into molecular pathogenesis, which will be of interest to a broader readership, e.g. researchers involved in AI, big data and systems-modelling. Below are papers that are relevant to that end as they rely on cell-type specific data in invertebrate and mouse models and as they focus on compensatory and homeostatic genes and pathways that may be commonly engaged in response to several types of neurodegenerative disease triggers.

Reply:

Thank you we appreciate this important note. In the current study we compared our dataset to 3 external datasets. However we feel that further comparison to mouse nd invertebrate models of neurodegenerative diseases would take out the focus from the paper. We did apply advanced bioinformatic statistical and computational data analyses on our data so our paper may be of interest to AI big data and system modelling researchers as is.

General comment: we formatted the references to PLoS ONE style and included the supporting table info after the acknowledgments, the funding and competing interests sections are included separately. We changed heading text size to 14.

We also wrote a new cover letter.
