# Supplementary material for: Single-cell RNA sequencing analysis of human Alzheimer’s disease brain samples reveals neuronal and glial specific cells differential expression
Source: PLoS One. 2023 Feb 24;18(2):e0277630. doi: 10.1371/journal.pone.0277630 (PMC9955959; doi:10.1371/journal.pone.0277630)
Supplement: Attachment — Submitted filename: Rebuttle letter.docx [file pone.0277630.s007.docx]

Rebuttal letter (PLoS ONE) Soreq et al (title, Single-cell RNA sequencing analysis of human Alzheimer’s disease brain samples reveals neuronal and glial specific cells differential expression) Oct. 2022

I corrected the paper according to the recent reviewer comments and kept ‘track changes’. I am also re submitting a version with no tracked changes.

Specifically, I addressed the following issues:

1. Correction of patient’s staging (BRAAK stages I/II)
2. No APOE/CERAD/THAL scores were taken for the patients or controls
3. The microglia showed increased (not decreased) expression, corrected
4. Slight correction of the paper title is included.
5. The included brain region in the paper analyses is the frontal cortex only (for simplicity).
6. Comparison to our previous exon array aging study but we felt that this comparison suffice and I added further details regarding the microglia depleted mice comparative study.

Sincerely, Dr.Lilach Soreq

UCL ION London UK
